# Neck Vascular Biomechanical Dysfunction Precedes Brain Biochemical Alterations in a Murine Model of Alzheimer's Disease

**DOI:** 10.1523/ENEURO.0293-25.2025

**Published:** 2026-02-03

**Authors:** Allison R. Jones, Amin Jarrahi, Kylee Karpowich, Lindsay P. Brown, Kalynn M. Schulz, Rebecca A. Prosser, A. Colleen Crouch

**Affiliations:** ^1^Department of Biomedical Engineering, University of Tennessee, Knoxville, Tennessee 37916; ^2^Department of Biochemistry and Molecular Biology, University of Tennessee, Knoxville, Tennessee 37916; ^3^Department of Chemistry, University of Tennessee, Knoxville, Tennessee 37916; ^4^Department of Psychology, University of Tennessee, Knoxville, Tennessee 37916

**Keywords:** blood–brain barrier, hippocampus, neurodegenerative diseases, transgenic mice, vascular remodeling

## Abstract

Age-related vascular changes accompany or precede the development of Alzheimer's disease (AD) pathology. The comorbidity of AD and arterial stiffening suggests that vascular changes have a pathogenic role. Carotid artery mechanics and hemodynamics have been associated with age-related cognitive decline. However, the impact of hemodynamics and vascular mechanics on regional vulnerability within the brain has not been thoroughly explored. Compared with the arterial system, brain venous circulation in cognitive impairment is less understood despite the venous system's role in transport. To study vasculature impact on biochemistry in AD models, we must first establish the differences in vasculature mechanics and hemodynamics in a common AD model compared with healthy controls. With this baseline data, future studies on manipulating vasculature integrity in mice become feasible. Young and aged female 3xTg mice and age-matched controls were imaged using a combination of ultrasound and mass spectrometry. Wall shear stress varied across age and AD models. Mean velocity and pulsatility index varied across age and AD. Liquid chromatography-mass spectrometry of brain tissue revealed several lipids that were statistically different between age and AD, and matrix-assisted laser desorption/ionization MS imaging revealed region-specific differences between groups. Combining both ultrasound and mass spectrometry, we were able to detect significant changes in the vascular biomechanics of neck vasculature prior to observing significant changes in the brain biochemistry. Our work revealed significant vascular differences in the 3xTg compared with controls and, to our knowledge, is the first to study vascular biomechanics via ultrasound in the 3xTg AD mouse model.

## Significance Statement

While carotid artery mechanics have been linked to cognitive decline, their contribution to region-specific brain vulnerability remains underexplored. This study establishes a crucial foundation by comparing neck vascular biomechanics with brain biochemistry between young and aged AD mice and age-matched controls. Using ultrasound imaging and mass spectrometry, we detected significant differences in wall shear stress, pulsatility index, and mean velocity. Vascular alterations were observed prior to significant biochemical changes in brain tissue, suggesting a mechanistic role of vasculature dysfunction in AD progression. Our findings provide evidence of altered neck vascular biomechanics in the 3xTg mouse model using ultrasound, emphasizing the potential of vascular imaging as an early diagnostic and therapeutic target in Alzheimer's disease.

## Introduction

In 2024, approximately 7 million people in the United States were living with Alzheimer’s disease (AD), and by 2060, this number is projected to nearly triple to 14 million (Alzheimer’s Association, 2025b). Alzheimer’s disease (AD) is the most common type of dementia and is responsible for progressive memory loss, cognitive dysfunction, and behavioral changes (Toledo et al., 2013). AD is currently a leading cause of death worldwide in part due to the lack of effective treatments (Cortes-Canteli and Iadecola, 2020). AD has been characterized by an accumulation of amyloid plaques and neurofibrillary tangles in the brain (Cortes-Canteli and Iadecola, 2020); however, current medications have not been shown to prevent onset or reverse the progression of cognitive decline. Currently, the only treatment available is designed to slow the progression of the disease by targeting amyloid plaques (Winder et al., 2021; Alzheimer’s Association, 2025a). To develop a treatment for AD, the causes of the disease must be thoroughly understood. One promising avenue of research is the connection between cardiovascular function and neurological disorders. In 2019, a meta-analysis showed that increased arterial stiffness is associated with markers of cerebral small-vessel disease and cognitive decline (Palta et al., 2019).

The comorbidity of AD and pathologies such as arterial stiffening may suggest that vascular changes have a pathogenic role (DuPont et al., 2019; Cortes-Canteli and Iadecola, 2020; Winder et al., 2021). Carotid artery mechanics [increased stiffness (Winder et al., 2021)] and hemodynamics [lower wall shear stress (WSS; Mutsaerts et al., 2011)] have been associated with age-related cognitive decline. However, the impact of hemodynamics and vascular mechanics on regional vulnerability within the brain has not been thoroughly explored. Downstream vascular effects, including increased jugular venous reflux, are associated with white matter disease and aging (Chung et al., 2014). Despite the venous system’s role in transport, the impact of age-related alterations of brain venous circulation on cognitive impairment and dementia is much less understood compared with the arterial system (Fulop et al., 2019). Additionally, vascular changes accompany or precede the development of AD’s pathology and could be used as a biomarker for age-related dementia (Kehmeier and Walker, 2021). The effect of vascular mechanics on cognitive function has only recently been explored clinically via ultrasound, but further investigation is needed to determine which vascular factors may serve as effective diagnostic indicators for AD (Zhang et al., 2025).

The molecular changes that lead to arterial stiffening and AD have been independently studied. Lipid alterations, in particular, have been linked to both arterial stiffening and AD (Thal et al., 2003; Tian et al., 2004; de Oliveira et al., 2017; Mulè et al., 2017; Arnett et al., 2019; Kao et al., 2020; Moerman et al., 2021; Kelley, 2022; Baba et al., 2023). Lipids are crucial for a wide range of biological functions, including energy storage, cellular structure, and signaling processes. Not only are lipids relevant in vascular dysfunction, but lipid dysregulation and composition are implicated in various neurological disorders (Amtul et al., 2011; Kim et al., 2015; Katsel and Haroutunian, 2019; Kao et al., 2020). The brain is one of the most lipid-rich organs, which makes understanding the lipids involved in brain-related diseases such as AD vital for understanding the disease itself.

To study vasculature impact on the brain biochemistry in AD models, we must first establish the differences in vasculature mechanics and hemodynamics in a common AD model compared with healthy controls. With this baseline data, we can complete future studies on manipulating vasculature integrity in the mice (Pezet et al., 2008; Stover et al., 2015; C. Zhang et al., 2024). One of the most popular mouse models is the 3xTg AD transgenic model that expresses a human tauopathy mutation (tauP301L) in addition to APP (K670N/M671L) and PS1 (M146V) mutations associated with amyloid pathology (The Jackson Laboratory; Oddo et al., 2003; Stover et al., 2015; Dennison et al., 2021). To study vasculature impact on biochemistry in AD models, we first established the baseline differences in vasculature mechanics and hemodynamics and brain lipidomics in common AD models compared with healthy controls across ages. With this baseline data, we can complete future studies that manipulate the underlying cardiovascular system in the mice.

In this work, we used ultrasound to assess the biomechanics of the neck vasculature, matrix-assisted laser desorption/ionization mass spectrometry imaging (MALDI MSI) to spatially resolve lipid changes in the brain, and liquid chromatography-mass spectrometry (LC-MS) to annotate the lipids by measuring their exact mass and fragmentation patterns. By combining ultrasound, MALDI mass spectrometry imaging, LC-MS, and histology data, we can begin to understand how aging and AD affect the relationship between the vasculature and the brain. With the growth of an aging population and the increasing burden of health care for individuals with AD, studying the causes of AD is vital to reduce morbidity and mortality (Xia et al., 2018).

## Materials and Methods

### Animal models

All animal work was approved by the Institutional Animal Care and Use Committee. *N* = 6 young (12 weeks) and *N* = 6 aged (52 weeks) female B6;129-Tg(APPSwe,tauP301L)1Lfa *Psen1^tm1Mpm^* (3xTg) mice and age-matched controls (The Jackson Laboratory) were used for this experiment (Oddo et al., 2003). The maturation rate of mice does not share a linear correlation with humans, so 12 weeks is ∼20 years old versus 52 weeks which is ∼45 years old (Crumpler et al., 2021). Female 3xTg mice were used because the males have been shown not to develop the phenotypic traits of Alzheimer’s disease (AD) (Dennison et al., 2021). Since women are more at risk of developing AD, with two-thirds of patients with AD being women, studying a female AD model will provide more insight into the disease (Zhu et al., 2021). The average weights are reported in Table 1 (*p* < 0.0001). The vasculature of the mice was imaged via ultrasound (Vevo 3100, VisualSonics). After conscious decapitation, the brains were removed for analysis with mass spectrometry.

**Table 1.** Mouse body mass

Mouse	Average mass (g)
YFM C^*♦^	23.0 ± 1.41
YFM AD^^∇^	22.8 ± 2.14
OFM C^*∇α^	45.2 ± 5.23
OFM AD^^♦α^	53.2 ± 7.63

The aged 3xTg and aged control were statistically significant with *p* < 0.0001. The symbols represent statistically significant differences between pairs.

### Ultrasound

Mice were anesthetized with isoflurane and then imaged via ultrasound using the Vevo 3100 with the MX550D: 55 MHz MX series transducer with a gain setting of 35 dB. Images were collected and analyzed with Vevo LAB (VisualSonics) in B-mode to measure the diameter of the carotid arteries and jugular veins. The diameter of the carotid artery was measured in three different locations directly inferior to the carotid bifurcation. The average diameter of the artery was calculated for both systole and diastole. The diameter of the jugular vein was also measured at three different time points across the cardiac cycle to provide an average diameter.

The blood velocity through the carotid and jugular was measured using pulsed wave (PW) Doppler mode during systole and diastole. For the carotid artery, three velocity peaks in systole across the cardiac cycle were measured and averaged to provide an average velocity, and the process was repeated in diastole. For the jugular vein, three velocity measurements were averaged to provide an average velocity. Using the velocity and diameter for the carotid in systole and diastole, the wall shear stress (WSS) was calculated. WSS was calculated using the following equation:

$$\begin{array}{c} \tau =\frac{8\mu \bar{u}}{d} \end{array},$$

where *µ* is the viscosity of blood, 0.035g/cm⋅s, u is the average velocity of blood, and d is the average diameter (Taylor et al., 1998; Greve et al., 2006; Wang et al., 2013; De Wilde et al., 2016). In previous studies, the Hagen–Poiseuille application (Vlachopoulos et al., 2011) has been used for the WSS measurements in large arteries although rigid walls are assumed. Since large arteries, such as the carotid artery, mimic the structure of a cylindrical tube, this technique is an appropriate method due to its simplicity (Wang et al., 2013; Crouch et al., 2020).

For the carotid arteries, the average volumetric blood flow, *V*_flow_, was calculated using the following equation:

$$\begin{array}{c} V_{\mathrm{flow}}=A\cdot V \end{array},$$

where *A* is the cross-sectional area of the vessel and *V* is the average velocity of blood. Vascular flow was normalized by individual body weight to account for differences in blood volume for larger animals.

Pulsatility index (PI) is a noninvasive method of measuring vascular resistance with higher values indicating greater pulsatile flow possibly indicating vascular stiffening (Wielicka et al., 2020). For the carotid arteries, the mean velocity was measured and used to calculate the pulsatility index (PI) using the following equation:

$$\begin{array}{c} \mathrm{PI}=\frac{\mathrm{PSV}-\mathrm{EDV}}{\mathrm{MV}} \end{array},$$

where PSV is the peak systolic velocity, EDV is the end diastolic velocity, and MV is the mean velocity across the cardiac cycle (Bill et al., 2023).

The values of the diameters were used to calculate the circumferential cyclic strain (CCS) (*E*) using the following equation:

$$\begin{array}{c} E_{\theta \theta }=\frac{1}{2}\left(\frac{D_{s}^{2}}{D_{d}^{2}}-1\right) \end{array},$$

where $D_{s}$ represents the diameter during systole and $D_{d}$ represents the diameter during diastole for circular vessels (Humphrey and Delange, 2004; Goergen et al., 2010; Crouch et al., 2018). CCS was not measured in the jugular veins because veins are generally low-pressure, low-flow vessels compared with the arteries, do not mimic cylindrical tubes, and do not typically pulsate in the same way (Palmer et al., 2017).

#### Statistical analysis

Using JMP, a two-way ANOVA and a Tukey’s HSD post hoc test were performed to determine statistical difference between age and AD versus control groups for circumferential cyclic strain (CCS), WSS in systole and diastole for the carotid, WSS for the jugular vein, and pulsatility index for the carotid. Data are reported as mean, interquartile range, standard error, and individual data points with significance set to *p* < 0.05.

### Tissue harvesting and preparation

After ultrasound, mice were euthanized via conscious decapitation for quick removal of the brain. Conscious decapitation does not require the use of an anesthetic, such as isoflurane, which can have an effect on the brain biochemistry (Baer et al., 2020). The brains were cut in half, and the right hemisphere of the brains was then flash frozen in liquid nitrogen by floating the tissue in aluminum foil boats. The brains were cryosectioned at 10 µm using a Leica Cryostat and mounted on positively charged glass microscope slides (Thermo Fisher Scientific) for MALDI MSI. The location of each brain slice was determined using a standard mouse brain atlas (Allen Brain Atlas, 2011), and samples were collected at a location approximately −2.24 ± 0.21 mm from the bregma. Serial sections (∼60 µm) were collected in tubes for LC-MS analysis. The slides and tubes were stored in a −80°C freezer before analysis.

### Mass spectrometry

#### MALDI MSI

2,5-Dihydroxybenzoic acid at 40 mg/ml in 70% MeOH was sprayed on the slides using an HTX M3+ sprayer. The matrix was sprayed on the tissue in a crisscross pattern 10 times at a temperature of 75°C and a pressure of 10 psi N_2_ gas. Sprayer parameters included a flow rate of 100 µm/min, velocity of 1,200 mm/min, track spacing of 3 mm, and a drying time of 10 s. Once the tissue was sprayed with the matrix, the slide was scanned (Epson scanner) and then placed in a Waters SYNAPT G2 time-of-flight mass spectrometer. Samples were run in positive mode with a pixel size set to 60 µm, 300 laser shots fired at 1 kHz using a laser pulse energy with an average of 25 µJ. The laser was adjusted to 300 arbitrary units (a.u.) (McDonald et al., 2023). The mass spectra were processed using MassLynx software, and the resulting molecular ion images were analyzed using HD Imaging (Waters Corporation). Regions of interest were manually segmented based on anatomical landmarks using a standard mouse brain atlas (Allen Brain Atlas, 2011).

#### LC-MS untargeted lipidomics

LC-MS was performed by the Biological and Small Molecule Mass Spectrometry Core (BSMMSC) at the University of Tennessee, Knoxville, to identify lipids and lipid-like metabolites in the brain samples. Samples were stored at −80°C prior to extraction, which was conducted following the Bligh and Dyer method (Bligh and Dyer, 1959) using HPLC grade reagents (Thermo Fisher Scientific). Briefly, isotopically labeled internal standard (Avanti Research) was added to the sample along with 750 µl of 1:2 chloroform:methanol and vortexed. Following mixing, 250 µl of chloroform and 250 µl of water were added, and the solution was vortexed and centrifuged for 5 min at 13,000 rpm. The lower layer (organic layer) was collected and added to a glass vial. The extraction was repeated with an additional 250 µl of chloroform, and the resulting organic layer was pooled into the glass vial. Following vortexing, the extract was aliquoted and dried under a stream of nitrogen. LC-MS analysis was performed using a Vanquish UHPLC coupled to an Exploris 120 mass spectrometer (Thermo Fisher Scientific). Lipids were separated on a Accucore C30 2.1 mm × 100 mm, 2.6 µm column using a gradient of mobile Phase A (60:40 water:acetonitrile with 10 mM ammonium formate and 0.1% formic acid) and mobile Phase B (90:10 isopropanol:acetonitrile with 10 mM ammonium formate and 0.1% formic acid) with the following gradient: 0 min 32% B; 1.5 min 32% B; 4 min 45% B; 5 min 52% B; 8 min 58% B; 11 min 66% B; 14 min 70% B; 18 min 75% B; 21 min 97% B; 25 min 97% B; 25.1 min 32% B; 30 min 32% B. The flow rate was set to 0.260 ml/min. Data were collected in both positive and negative mode using data-dependent acquisition at a resolution of 120,000. Feature-finding and lipid annotation were conducted using MS-Dial (Version 4.9) (Tsugawa et al., 2015). Raw area counts of annotated lipids in positive and negative mode were normalized by the internal standard and further processed using an in-house Python script where normalized values were log transformed. Individual lipids annotated from the LC-MS untargeted lipidomics analysis were cross-referenced with those found from the MALDI MSI analysis for tentative lipid annotations for MALDI MSI. Lipid annotations and abbreviations can be found in Extended Data Figure 4-1.

#### Statistical analysis

Statistical changes in lipid headgroup composition were evaluated using normalized/log-transformed LC-MS data from untargeted lipidomics. In each sample, lipids pertaining to a specific headgroup were summed, and a *t* test (two-tailed distribution, homoscedastic) was performed to evaluate statistical significance between the two compared groups. Fold changes were also calculated to illustrate the differential abundance of lipid headgroups for each comparison. MetaboAnalyst (Version 5.0) (Pang et al., 2021) was used to perform a partial least squares discriminant analysis (PLSDA) on tested groups via one-factor statistical analysis. For MALDI MSI, a Pearson’s correlation coefficient analysis was run in HD Imaging to identify spatial correlation between mass spectra within a sample (*R* > 0.6).

### Staining

After MALDI MSI, the slides were placed in a desiccator prior to staining. The matrix was removed using ethanol and HPLC water. After removing the matrix, the slide was air-dried for 5 min before beginning the staining protocol (Methanol Fixation, H&E Staining & Imaging for Visium Spatial Protocols Overview, 2024). The slide was coated with isopropanol for 1 min. Isopropanol was removed, and the slide was coated in hematoxylin for 7 min. The hematoxylin was removed, and the slide was submerged in HPLC water. The slide was then coated in bluing buffer for 2 min and then submerged in HPLC water. Eosin mix was prepared using a combination of tris-acetic acid and Eosin Y. The slide was coated in the eosin mix for 1 min and then submerged in HPLC water. The slide was placed in the thermocycler for 5 min to dry. A coverslip was placed on the slide to prepare for imaging with the Leica M205 FCA microscope.

## Results

### Ultrasound

**Figure 1.**
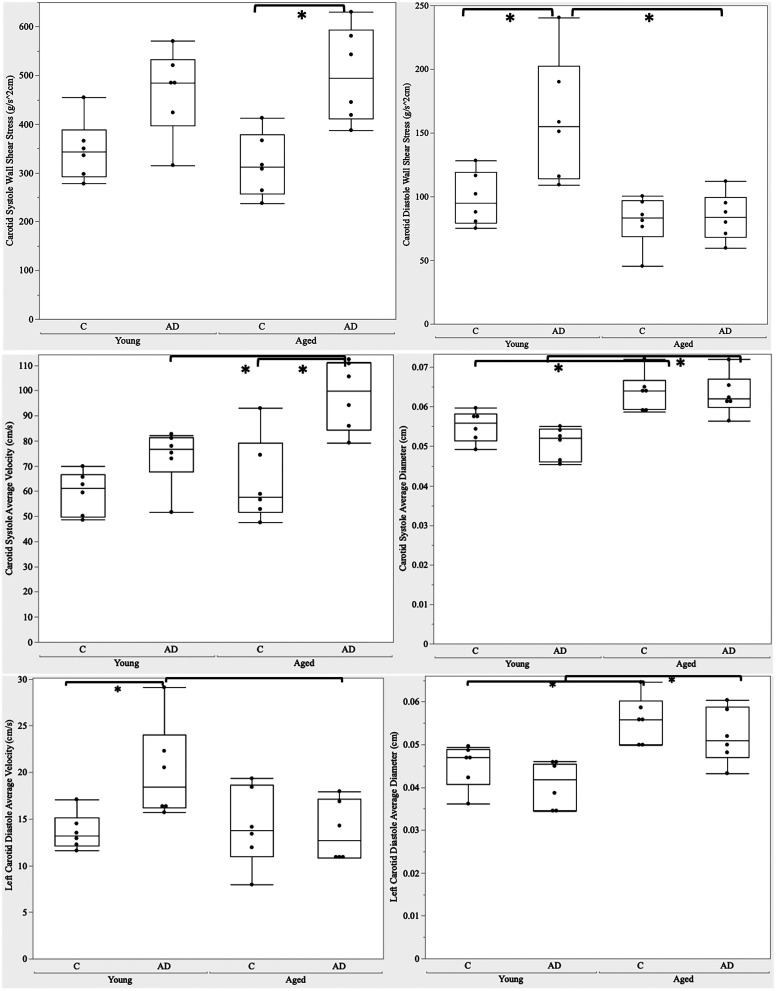
Wall shear stress of the common carotid artery during (top left) systole and (top right) diastole. Wall shear stress varied across age and AD with significance *p* < 0.05 denoted between groups (*). Young (12 weeks), aged (12 months); C, control; AD, 3xTg Alzheimer model. Bottom left, Average velocity of the common carotid artery during systole. Bottom right, Average diameter of the common carotid artery during systole. Diameter and velocity varied with respect to age and AD with significance *p* < 0.05 denoted between groups (*).

The WSS of the common carotid artery (CCA) in systole (*p* = 0.001) and diastole (*p* = 0.0004) were statistically significant between groups, as shown in Figure 1. During systole, disease had the largest effect (*p* = 0.0001) with aged 3xTg WSS increased by 57.7% compared with aged controls (*p* = 0.004). Without the presence of AD, WSS did not significantly change with age.

10.1523/ENEURO.0293-25.2025.f1-1 Figure 1-1 ANOVA and post-hoc p-values of CCA diameter and velocity measurements from ultrasound data. Download Figure 1-1, DOCX file.

Aged 3xTg (OAD) versus young controls (YC, *p* = 0.02) and young AD (YAD) versus aged controls (OC, *p* = 0.02) were also statistically significant. Although not statistically significant, young 3xTg WSS trended toward an increase of ∼30% compared with young controls. For diastole, age (*p* = 0.001), disease state (*p* = 0.01), and the combination of age and disease (*p* = 0.02) had an effect on WSS. During diastole, young 3xTg WSS was increased by 63.5% compared with young controls (*p* = 0.008). Aged 3xTg WSS decreased by 47.6% compared with young 3xTg (*p* = 0.001). Young AD versus aged control was also statistically significant (*p* = 0.0008). As seen in Figure 1, the increase in WSS of the carotid during systole between aged controls and aged 3xTg was driven by a significant increase in average velocity during systole. Similarly for diastole in the young groups, velocity significantly increased for young AD versus controls; conversely, for the AD groups, changes in diastolic WSS were driven by the increase in vessel wall diameter and the decrease in average velocity in the aged AD group versus the young AD group. ANOVA and post hoc *p*-values can be found in Extended Data Figure 1-1.

**Figure 2.**
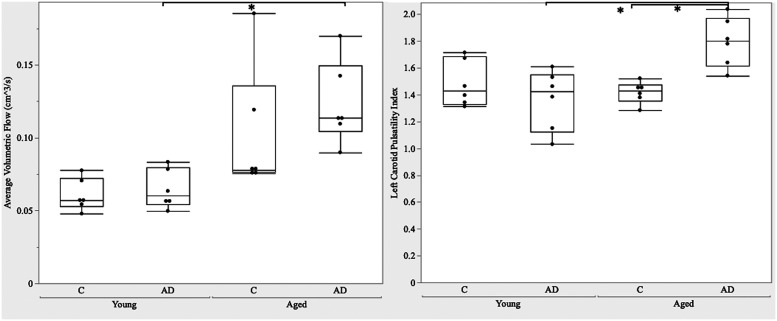
Left, Average volumetric flow of the common carotid artery (CCA) over the cardiac cycle. Right, Pulsatility index of the CCA. Average volumetric flow varied across age with significance *p* < 0.05 denoted by (*). PI varied across age and AD with significance of *p* < 0.05 denoted between groups (*).

The volumetric flow values of the CCA were statistically significant between groups (*p* = 0.002), as shown in Figure 2. Age (*p* = 0.003) had a significant effect on volumetric blood flow. Volumetric flow increased 50.4% in aged 3xTg compared with young control (*p* = 0.0045) and increased 47.9% in aged 3xTg compared with young 3xTg (*p* = 0.0078). When volumetric flow was normalized to body weight, flow decreased by 22.7% in the aged 3xTg group compared with the young 3xTg group.

The pulsatility index of the CCA was statistically significant between groups (*p* = 0.002), as shown in Figure 2. Age (*p* = 0.02) and the combination of age and disease (*p* = 0.002) had effects on pulsatility index. Pulsatility index increased by 31.6% in aged 3xTg compared with young 3xTg (*p* = 0.002) and increased by 26.1% in aged 3xTg compared with aged controls (*p* = 0.006). Aged AD and young controls were also statistically significant. Our results indicate a significant increase in pulsatility index with the simultaneous presence of AD and aging.

The CCS values for the carotid arteries and the WSS values for the jugular veins between groups were measured but were not statistically significant. For the carotid, the CCS was 26.1 ± 12.3 for young controls, 30.2 ± 16.8 for young 3xTg, 16.2 ± 8.85 for old controls, and 25.0 ± 12.6 for aged 3xTg. For the right carotid, the CCS was 23.0 ± 7.93 for young controls, 30.5 ± 15.8 for young 3xTg, 22.8 ± 4.91 for aged controls, and 31.0 ± 12.4 for aged 3xTg.

### Mass spectrometry

**Figure 3.**
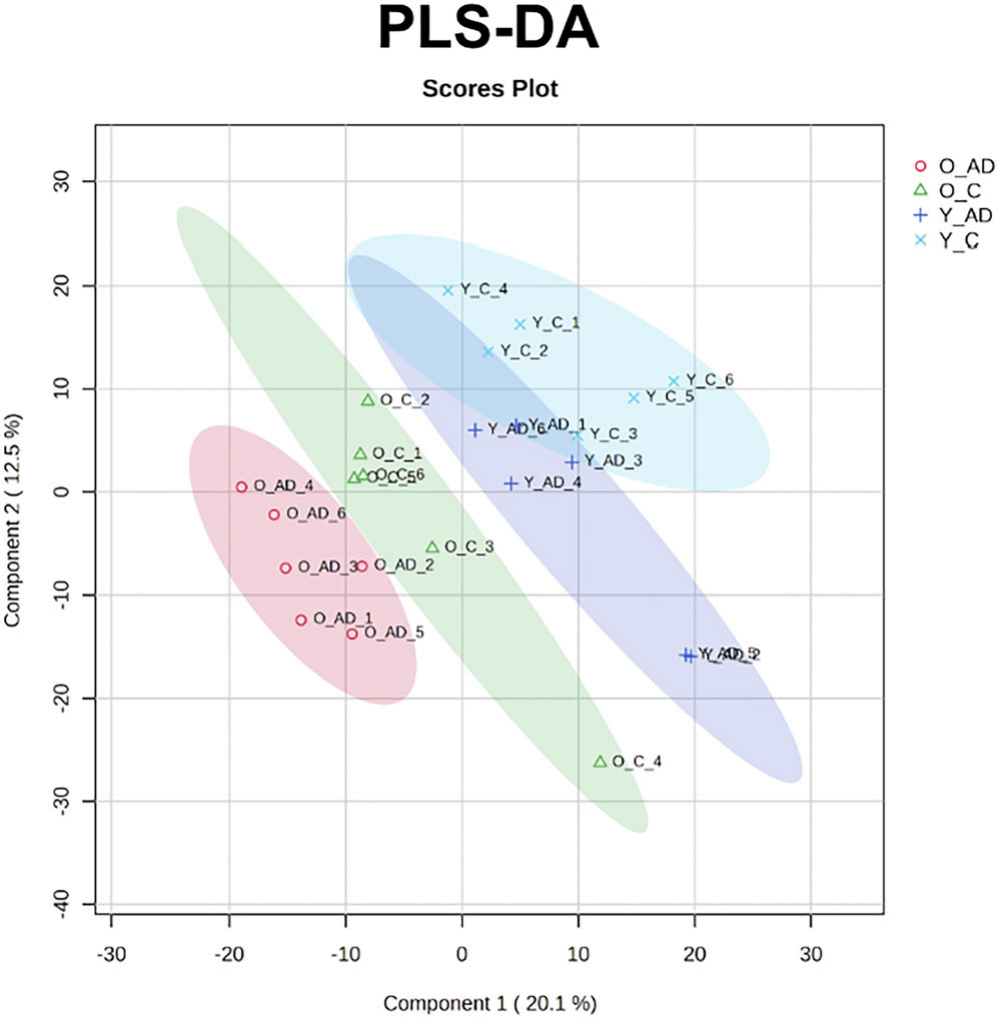
PLSDA of all four groups which emphasizes the variations between all four groups with greater separation between aged AD and aged control.

**Figure 4.**
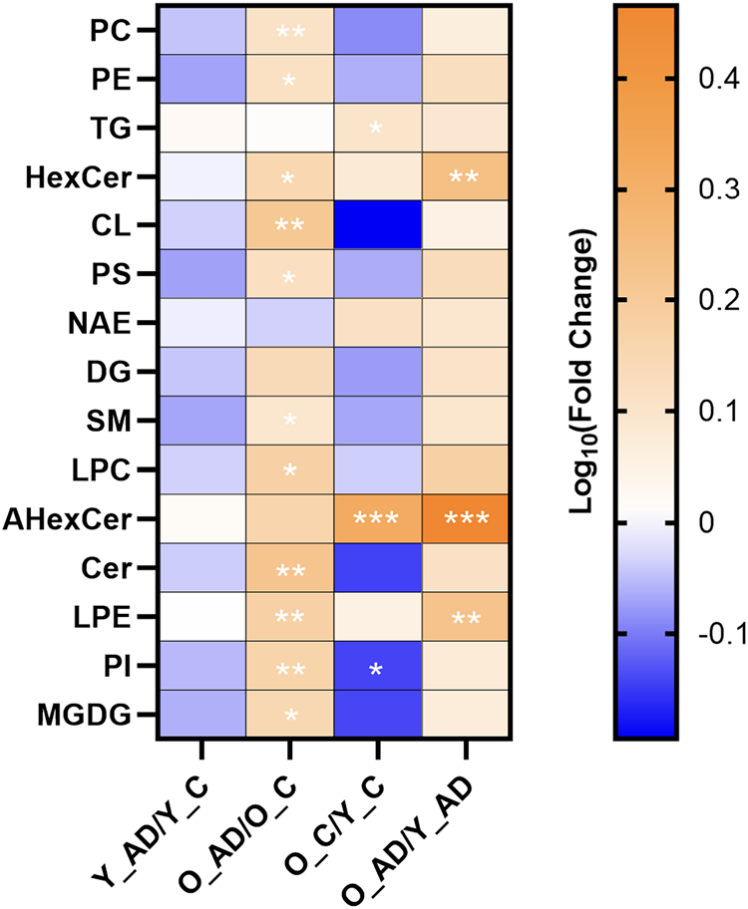
Heatmap of lipid headgroups with 10 or more species detected in LC-MS brain samples. The heatmap provides a visual representation of the fold changes between the four groups listed at the bottom, with significance of *p* < 0.1 (*), *p* < 0.05 (**), and *p* < 0.01 (***) denoted between groups.

LC-MS results identified several lipids with statistically significant quantities (*p* < 0.05). A PLSDA was performed on LC-MS data as shown in Figure 3, revealing statistical separation of all groups tested. The abundance of several lipid headgroups changed significantly with respect to age and AD genotype, as shown in Figure 4. For headgroup analysis, the most significant changes in lipid headgroup composition were seen when comparing the aged AD group with the aged control group. There were no significant changes seen in the young AD group compared with the young control group.

10.1523/ENEURO.0293-25.2025.f4-1 Figure 4-1 List of lipid classes and their respective abbreviations. Download Figure 4-1, DOCX file.

**Figure 5.**
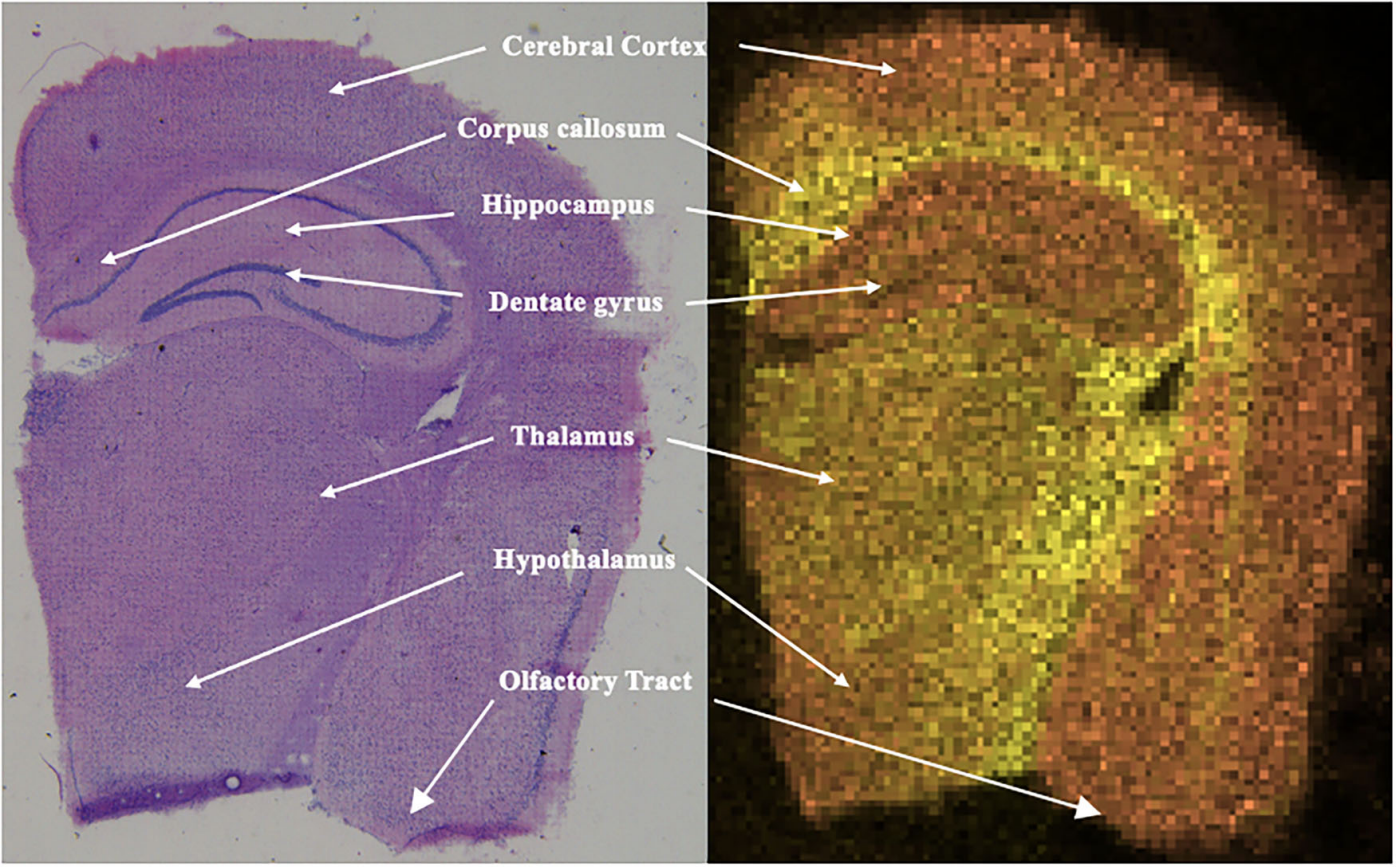
Right, H&E-stained microscope image of the right hemisphere of an aged 3xTg brain tissue. Left, Molecular overlay of two different molecules in the same aged 3xTg brain tissue. PC O-(37:9), a phosphatidylcholine with an *m*/*z* of 772.53, is depicted in orange, and PC 40:4, with an *m*/*z* of 838.62, is depicted in yellow. This image shows the localization of some of the most intense lipids found in the brain tissue.

**Figure 6.**
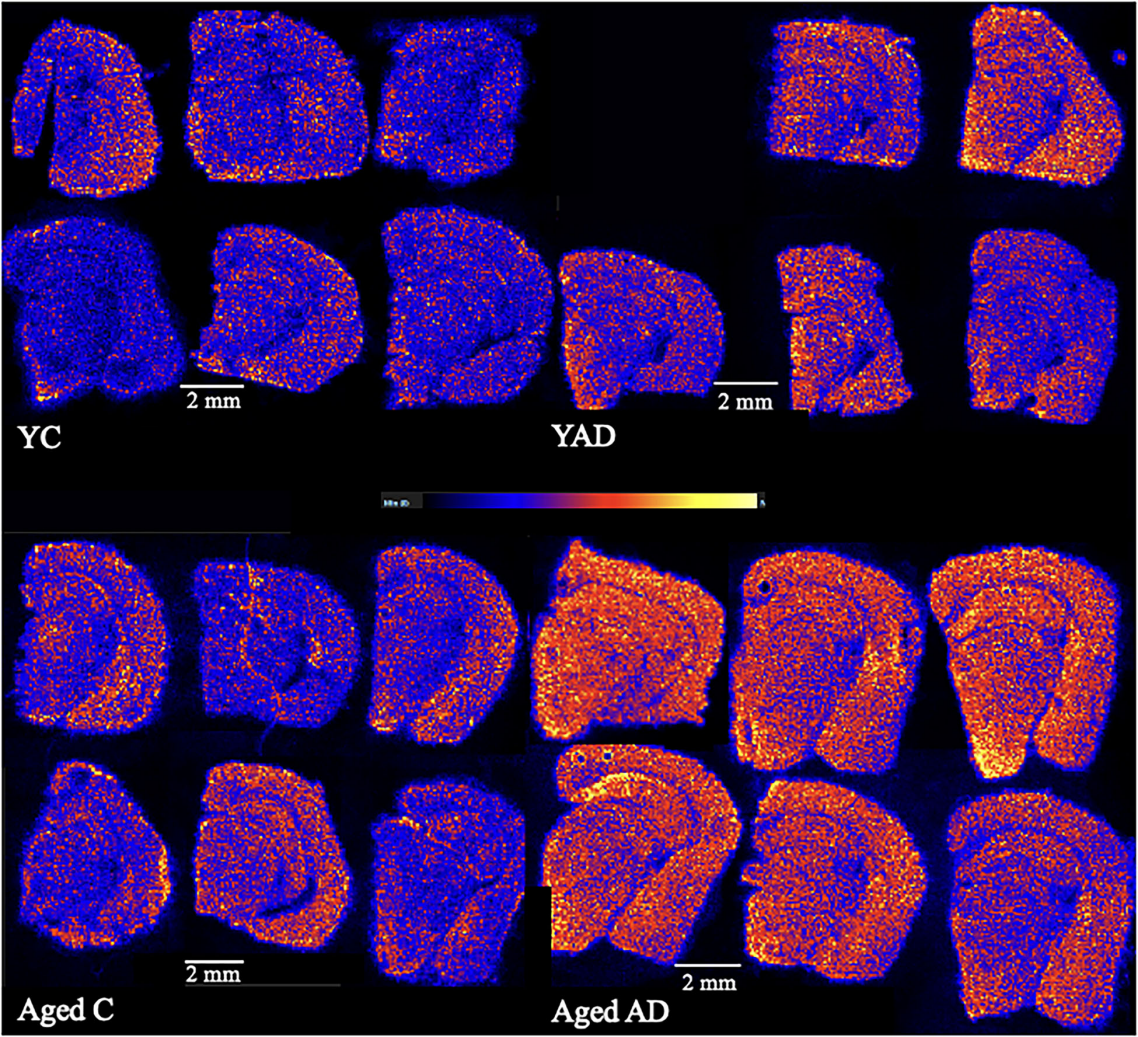
PE O-42:10, with an *m*/*z* of 798.54, present in the brain tissue of all four groups. Images show intensity and localization of PE O-(42:10) in the brain. Top left, Young control 1–6. Top right, Young 3xTg 2–6 (YAD 1 not pictured due to issue during MALDI MSI data collection). Bottom left, Aged control 1–6. Bottom right, Aged 3xTg 1–6.

To demonstrate the distinct lipid composition in various parts of the brain that can be studied with MSI, PC O-(37:9), a phosphatidylcholine with an *m*/*z* of 772.53, is depicted in orange in Figure 5 and is primarily located in the hippocampus and outer region of the brain compared with PC 40:4 shown in yellow. From the LC-MS results, PC O-(37:9) was found to be more abundant in aged 3xTg compared with aged controls with a fold change of 1.96 (*p* = 0.007). Visually with MALDI MSI, PC O-(37:9) was found in all four groups and was within the highest average intensities across the entire tissue sample. PE O-(42:10) (*m*/*z* = 798.54) is significantly increased in AD compared with control, and MALDI MSI revealed that this lipid is in all regions of the brain slice for aged AD as shown in Figure 6. PE O-(42:10) was among the top five most intense lipids observed across all six aged 3xTg mice.

**Figure 7.**
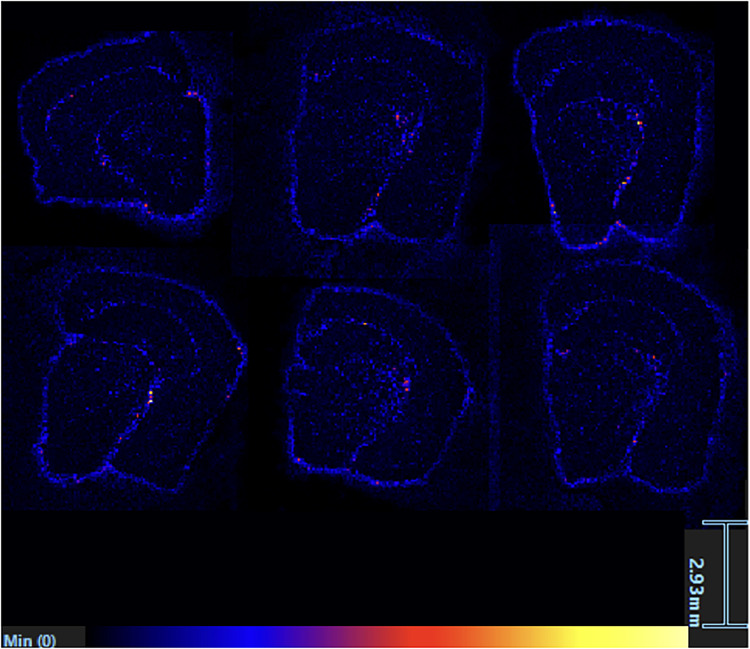
LPC 16:0 [M + H]+, with an *m*/*z* of 496.34, present in all six aged 3xTg brain tissue. These images show the localization of this lipid within the right hemisphere of each brain starting with OFM1-OFM3 from left to right in the top row and OFM4-OFM6 from left to right in the bottom row. LPC 16:0 [M + H]+ can be seen primarily in the outer region of the hippocampus, a region vital to the detection of Alzheimer’s disease.

**Figure 8.**
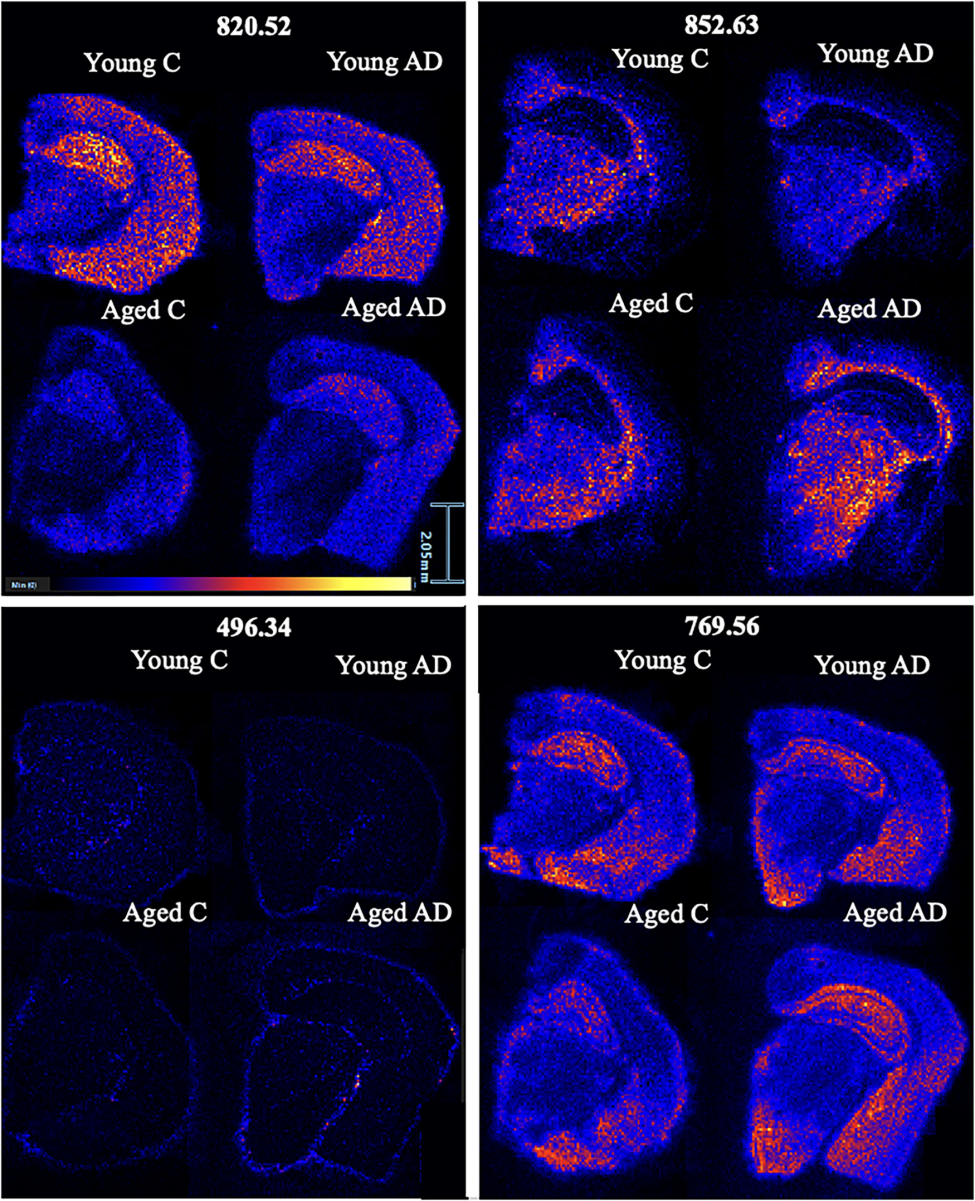
Representative image from each of the four groups showing the spatial distribution of specific lipids within the brain tissue. Top left, HexCer 16:3 with an *m*/*z* of 852.63. Top right, A PC with an *m*/*z* of 820.52. Bottom left, LPC 16:0 with an *m*/*z* of 496.34. Bottom right, SM 36:1;O2 with an *m*/*z* of 769.56.

From the LC-MS results, LPC 16:0 was more abundant in aged 3xTg compared with aged controls with a fold change of 1.41. Figure 7 shows the location and intensity of this lipid that was found in all six of the aged 3xTg group. LPE 18:1, a lysophosphatidylethanolamine with an *m*/*z* of 522.35, was more abundant in aged 3xTg compared with young 3xTg with a fold change of 4.68 (*p* = 0.0001), more abundant in aged controls compared with young controls with a fold change of 4.69 (*p* = 7 × 10^−9^), and more abundant in aged 3xTg compared with aged controls with a fold change of 1.47 (*p* = 0.02). Table 2 depicts a list of *m*/*z* values found in all six samples within all four groups and their corresponding correlated peaks based on spatial distribution. The correlated peaks were found to be similar in both their location and intensity (*R* > 0.6). Figure 8 shows a representative image from each of the four groups emphasizing the changes in the spatial distribution of specific lipids with respect to age and AD.

**Table 2.** List of *m*/*z* values with significant changes between groups and their correlated *m*/*z* values based on location and intensity across the tissue

*m*/*z*	Headgroup	Correlated *m*/*z*	Headgroup	Average *R*-value			
				Young C	Young AD	Aged C	Aged AD
772.53	PC	800.55	PC	0.97	0.95	0.96	0.95
		798.54	PE	0.97	0.95	0.95	0.95
		830.51	PC	0.9	0.87	0.9	0.87
798.54	PE	800.55	PC	0.99	0.98	0.98	0.98
		820.52	PC	0.94	0.87	0.91	0.89
		806.5	PC	0.62	0.87	0.74	0.88
		830.51	PS	0.9	0.89	0.91	0.88
496.34	LPC	524.37	LPC	0.84	0.86	0.79	0.86
		522.35	LPC	0.79	0.74	0.68	0.74
		554.28	PC	0.75	0.73	0.61	0.73
		518.32	LPC	0.74	0.71	0.6	0.71
524.37	LPC	496.34	LPC	0.84	0.86	0.79	0.86
		522.35	LPC	0.74	0.62	0.67	0.62

Values were selected for *R* > 0.6.

## Discussion

### Trends in vascular hemodynamic changes precede brain biochemistry alterations

Pulsatility index has been shown to increase with age and AD in both clinical patients and mice (Rivera-Rivera et al., 2016; Bill et al., 2023). Higher pulsatility index has been associated with increased vascular resistance and hypertension (Sasaki et al., 2021). The increase in pulsatility index seen in the aged AD group compared with aged controls indicates the potential presence of vascular dysfunction in the AD group.

With the presence of AD in the young group, significant changes in WSS were present; however, there were no significant changes in the lipid abundances in the brain (Table 3). These results indicate that at young ages, biomarkers for cognitive decline may not be detected in the brain, but the vasculature is already undergoing hemodynamic and biomechanical changes. The vascular and biochemical changes were significantly altered when comparing aged AD with young AD but remained unchanged when comparing aged control with young control. These results indicate that the presence of AD exacerbates vascular and biochemical changes seen with aging alone. Since significant vascular changes were observed prior to changes seen in the brain biochemistry, it may be vital to more closely examine the vasculature as a biomarker for age-related dementia. Zhang et al. (2025) recently studied the role of vascular factors in AD patients, specifically via ultrasound imaging of the CCA. Their findings indicate that vascular factors that can be measured via ultrasound may contribute to the development of AD pathology.

**Table 3.** Relationship between trends seen in the ultrasound data and compared with trends found in the LC-MS data

Group	WSS (systole)	WSS (diastole)	Average velocity	PI	Lipid headgroups (↑)	Lipid headgroups (↓)
OC/YC (Age)	NS	NS	NS	NS	AHexCer, TG	PI
YAD/YC (AD)	NS (↑)	↓	NS (↑)	NS	NS	NS
OAD/OC (AD and age)	↓	NS	↓	↓	MGDG, PI, LPE, Cer, LPC, SM, PS, CL, HexCer, PE, PC	NS
OAD/YAD (AD and age)	NS	↓	NS (↑)	↑	LPE, AHexCer, HexCer	NS

NS, not statistically significant.

CCS has been shown to decrease with signs of vascular stiffening; however, there was no significant change from our ultrasound results (Park et al., 2012). The observed trend seen in CCS was a decrease with age but an increase with AD. In previous studies, WSS has been shown to decrease with age in both humans and mice causing endothelial dysfunction and arterial stiffness (Kamimura et al., 2022; He et al., 2024). The increase in WSS of the carotid during systole between aged controls and aged 3xTg was driven by a significant increase in average velocity during systole. An increase in PSV has been shown to increase with signs of cognitive impairment in clinical patients (Zhang et al., 2025). This supports our hypothesis that vascular stiffening, which leads to an increase in velocity in the large arteries, may contribute to AD. Since the large arteries lead directly to the microvasculature surrounding the brain, any fluctuation in blood flow can greatly impact the microvasculature and, therefore, lead to a disruption in the blood–brain barrier.

Age-related arterial stiffness can cause increased flow pulsatility in the microvasculature, potentially leading to cognitive decline and AD (Cooper and Mitchell, 2016). The microvasculature that consists of thin-walled arteries, arterioles, and capillaries is not designed to withstand high velocities, increasing the risk of damage. Since the brain is highly susceptible to fluctuations in blood pressure, the increase in velocity caused by vascular stiffening can have a significant effect, possibly leading to damage of vital regions in the brain (Winder et al., 2021). Over time, the constant high velocity and pressure in the cerebral arteries could lead to endothelial dysfunction and breakdown of the blood–brain barrier initiating cognitive decline indicative of AD (Kapasi and Schneider, 2016; van den Kerkhof et al., 2024).

### Body weight increases mediate vascular changes

The average mass between each of the four groups was statistically significant. Body mass significantly increased in the aged AD group compared with the age control group and in the aged AD group compared with the young AD group. Average volumetric flow significantly increased when comparing aged AD with young AD, however, when normalized to body weight, volumetric flow decreased by 20%. Lack of blood flow has been shown to correlate with AD by several other groups indicating vascular dysfunction (Roher et al., 2012; Crumpler et al., 2021).

### MALDI MSI reveals spatially relevant changes in the hippocampus with aging and AD

PCs and SMs, which play a role in cell signaling and are the primary components of neuronal cell membranes, were both elevated in the aged AD group compared with aged controls (Grimm et al., 2011). Since lipids are the mediators that manage many immune responses, they are vital when studying a disease such as AD, which may be associated with divergent immune responses (Katsel and Haroutunian, 2019). Lipids indicative of AD tend to be localized in different regions of the brain, which can be visualized using MALDI MSI data (Kao et al., 2020). PEs, which are involved in organ homeostasis, cell formation and maintenance, and signal transduction, were elevated in the aged AD compared with aged controls (Dimache et al., 2021). TGs play a role in the storage and transport of energy and were elevated in the aged control compared with the young control (Yoon et al., 2022). AHexCer, which is vital for cell function and structural components of cell membranes, was elevated in aged control compared with young control and aged AD compared with young AD (Fitzner et al., 2020). Ceramides play a role in signaling pathways and neurodevelopment and were elevated in aged AD compared with aged controls (Pant et al., 2020). LPEs, which are vital for neuronal growth and protection against cell damage, were elevated in aged AD compared with aged controls and aged AD compared with young AD (Kunii et al., 2021). HexCer, which is a structural component of myelin, was elevated in the aged AD group compared with the aged control group and in the aged AD group compared with the young AD group (Fitzner et al., 2020). CLs, which regulate metabolic processes, support mitochondrial functions, and promote brain cell viability, were elevated in the aged AD group compared with the aged control group (Ma et al., 2022). PS is involved in neuroinflammation, membrane signaling pathways, and neurotransmission and was elevated in the aged AD group compared with the aged control group. LPCs transport long-chain fatty acids to the brain, cell signaling, and remodeling of cell membranes and were elevated in the aged AD compared with the aged controls (Pant et al., 2020). LPC 18:0 with an *m*/*z* of 524.37 was found in all six aged 3xTg in both the LC-MS and MALDI MSI data and has been shown to increase with signs of plaque formation in atherosclerosis and AD (Ahsanul Haque et al., 2023; Cao et al., 2024). LPE 18:1 was found in all six aged 3xTg and has been associated with a quicker progression from mild cognitive impairment (MCI) to AD (Llano and Devanarayan, 2021). PIs make up the cell membrane of neurons and function as messengers for neurotransmitter signaling and were increased in aged AD compared with aged controls but decreased in aged controls compared with young controls (Kunii et al., 2021). MGDGs, which play a role in the development and function of myelin, were elevated in the aged AD group compared with the aged control (Blusztajn et al., 2023).

#### LPC 16:0 in control groups is distributed throughout the brain regions, but in AD the lipid is localized to the olfactory tract and cerebral cortex

LPC 16:0 was elevated in the aged 3xTg group compared with the aged control group and was shown in all six aged 3xTg brain tissue, primarily localized in the olfactory tract region that plays a crucial role in our sense of smell (Hintiryan et al., 2012). Loss of smell has been shown to have a positive correlation with signs of cognitive impairment indicative of AD (Tian et al., 2022). LPCs play an important role in inflammatory response, and the localization of LPC 16:0 in the olfactory tract may contribute to inflammation affecting our sense of smell (Leon et al., 2024; Leontyev et al., 2024). LPC 16:0 was also found to be uniformly distributed throughout the entire brain tissue in the control groups. However, in the AD groups, LPC 16:0 was primarily concentrated in the outermost regions of the brain, specifically in the olfactory tract.

### Consistent mouse model results with inconsistent clinical data show the need for better translatability between mouse models and clinical applications

There were several lipids that aligned with the results of previous studies both in human and rodent models (Tables 4 and 5). Most of the inconsistent findings are most likely due to variation in the genetic background and pathology of the model studied, highlighting the need to have baseline molecular characterization of AD models before studying novel treatments or comparing them with humans. The studies referenced provide context for the comparison of lipidomic trends rather than to imply direct mechanistic equivalence. Our findings indicate that the abundance and location of certain lipids significantly varied with age and AD.

**Table 4.** Comparison of lipid abundances between AD and age-matched control groups relative to results found by previous studies

Lipid	*m*/*z*	Group	Consistent	Model	Inconsistent	Model
SHexCer 42:1;O3	908.648	OAD > OC			OAD < OC (Q. Zhang et al., 2024)	Mouse
PC 32:0	756.55	OAD < OC	OAD < OC (Sabogal-Guáqueta et al., 2018)	Rats		
			OAD < OC (Whiley et al., 2014)	Clinical		
			OAD < OC (Zhang et al., 2021)	Mouse		
SM 36:1	753.58	OAD < OC			OAD > OC. (Koal et al., 2015)	Clinical
PC O 36:3	770.607	OAD > OC	OAD > OC (Puris et al., 2022)	Mouse		
TG 56:8	920.773	OAD < OC	MCI and AD < control (Bernath et al., 2020)	Clinical		
Cer 22:0	370.332	OAD < OC			MCI > control (Wackerlig et al., 2020)	Clinical
PC 38:1	816.648	OAD > OC	MCI > control (Abdullah et al., 2012)	Mouse		
PC 40:4	838.63	OAD > OC	MCI > control (Abdullah et al., 2012)	Mouse		
			AD > control (Proitsi et al., 2017)	Clinical		
PC 38:6	806.568	OAD > OC			OAD < OC (Whiley et al., 2014)	Clinical
PC 40:6	834.599	OAD > OC OC < YC			OAD < OC (Whiley et al., 2014)	Clinical
Cer 44:2	708.651	OAD > OC			MCI > Control (Marković et al., 2024)	Clinical
PE O-42:10	798.54	OAD > OC OC < YC	AD > control (Fernandes et al., 2024)	Cells		

**Table 5.** Comparison of lipid abundances between young and aged groups relative to results found by previous studies

Lipid	*m*/*z*	Group	Consistent	Model	Inconsistent	Model
Cer 22:0 O2	404.33	OAD > YAD	Old > young (Crivelli et al., 2024)	5xFAD		
Cer 22:3 O3	370.332	OAD > YAD OC > YC	Old > young (Crivelli et al., 2024)	5xFAD		
Cer 20:0	344.315	OAD > YAD	Old > young (Crivelli et al., 2024)	5xFAD		
PC O 36:3	770.607	OAD > YAD	Old > young (Panda et al., 2024)	C57BL/6		
DG 38:4	662.573	OAD > YAD	Old > young (Panda et al., 2024)	C57BL/6		
PC 38:3	814.55	OC < YC			Old > young (Panda et al., 2024)	C57BL/6
HexCer 40:1	800.661	OC > YC OAD > YAD	Old > young (Panda et al., 2024)	C57BL/6		

A few limitations were present in this study. First, using mouse models to study a disease present in humans such as AD creates some limitations since they are not biologically identical to humans. In clinical studies, LPC, PE, and PI, classes of phospholipids, have been shown to decrease with AD (Zubenko et al., 1999; Grimm et al., 2011; Knuplez and Marsche, 2020; Kalia et al., 2023; Su et al., 2024). In mouse studies, CLs have been shown to decrease with AD (Monteiro-Cardoso et al., 2015; Su et al., 2024). In clinical and mouse studies, PCs were shown to decrease with AD (Grimm et al., 2011). However, we observed a statistically significant increase in LPCs, PEs, CLs, and PCs in the aged 3xTG mouse model compared with aged controls. In mouse studies, LPC and PS, classes of phospholipids, have been shown to increase with AD in a previous study (Monteiro-Cardoso et al., 2015). In clinical studies, HexCer, CER, and SM, classes of sphingolipids; LPE, a class of phospholipids; and MGDG, a class of glycerolipids, have been shown to increase with AD in previous studies (Mielke et al., 2010; Torretta et al., 2018; Pujol-Lereis, 2019; Llano and Devanarayan, 2021; Su et al., 2024), which aligns with the statistically significant increase of LPC, HexCer, CER, SM, LPE, and MGDG observed in the aged 3xTg group compared with controls. Although another limitation of this study is that the age groups studied do not reflect older humans, the mouse model shows deficits by 12 months showing an already accelerated degeneration compared with humans. In the 3xTg mouse model, signs of cognitive decline have been detected as early as 3–5 months of age with the first signs of associative learning deficits (Roda et al., 2020). At ∼6 months of age, impairments in spatial working memory begin followed by deficits in recognition memory at ∼9–11 months. At ∼12 months, reference memory impairment is detected (Roda et al., 2020). More significant vascular changes are expected to occur in older, healthy control mice that represent older humans. Additionally, the ultrasound measurements were performed with the animals under anesthesia using isoflurane, which can potentially reduce signs of arterial stiffening (Constantinides et al., 2011).

In conclusion, our work emphasizes the differences in vascular biomechanics and brain biochemistry seen with aging and AD. Our ultrasound results indicate significant changes in WSS, PI, volumetric flow, velocity, and diameter of the carotid arteries with the presence of AD prior to changes observed in the brain biochemistry. From our LC-MS analysis, several lipids were significantly changed with the presence of aging and AD. MALDI MSI results were able to identify the spatial distribution and intensities of lipids annotated with LC-MS across the brain tissue. Overall, our study emphasized that vascular changes assessed by ultrasound imaging reveal markers of AD before biochemical lipid changes observed via mass spectrometry.

## Synthesis

Reviewing Editor: Timothy Jarome, Virginia Polytechnic Institute and State University Decisions are customarily a result of the Reviewing Editor and the peer reviewers coming together and discussing their recommendations until a consensus is reached. When revisions are invited, a fact-based synthesis statement explaining their decision and outlining what is needed to prepare a revision will be listed below. The following reviewer(s) agreed to reveal their identity: NONE. Note: If this manuscript was transferred from JNeurosci and a decision was made to accept the manuscript without peer review, a synthesis may not be available.

N/A
