# Supplementary material for: Neck Vascular Biomechanical Dysfunction Precedes Brain Biochemical Alterations in a Murine Model of Alzheimer's Disease
Source: eNeuro. 2026 Feb 3;13(2):ENEURO.0293-25.2025. doi: 10.1523/ENEURO.0293-25.2025 (PMC12872301; doi:10.1523/ENEURO.0293-25.2025)
Supplement: Figure 1-1 — ANOVA and post-hoc p-values of CCA diameter and velocity measurements from ultrasound data. Download Figure 1-1, DOCX file. [file eneuro-13-ENEURO.0293-25.2025-s001.docx]

Figure 1-1: ANOVA and post-hoc p-values of CCA diameter and velocity measurements from ultrasound data.

|  | WSS (Systole) | WSS (Diastole) | Average Velocity (Systole) | Average Diameter (Systole) | Average Velocity (Diastole) | Average Diameter (Diastole) |
| --- | --- | --- | --- | --- | --- | --- |
| Total | 0.0014* | 0.0004* | 0.0002* | 0.0001* | 0.0222* | 0.0006* |
| YC/YAD | 0.0737 | 0.0083* | 0.2601 | 0.3804 | 0.0407* | 0.5357 |
| YAD/OAD | 0.8782 | 0.0013* | 0.0188* | 0.0007* | 0.0406* | 0.0111* |
| YC/OC | 0.9182 | 0.7394 | 0.9299 | 0.0143* | 0.9943 | 0.0169* |
| OC/OAD | 0.0037* | 0.9971 | 0.0010* | 0.9908 | 0.9942 | 0.6492 |
