# Supplementary material for: Neck Vascular Biomechanical Dysfunction Precedes Brain Biochemical Alterations in a Murine Model of Alzheimer's Disease
Source: eNeuro. 2026 Feb 3;13(2):ENEURO.0293-25.2025. doi: 10.1523/ENEURO.0293-25.2025 (PMC12872301; doi:10.1523/ENEURO.0293-25.2025)
Supplement: Figure 4-1 — List of lipid classes and their respective abbreviations. Download Figure 4-1, DOCX file. [file eneuro-13-ENEURO.0293-25.2025-s002.docx]

Figure 4-1: List of lipid classes and their respective abbreviations.

| **Abbreviation** | **Lipid class** |
| --- | --- |
| CAR | Acylcarnitines |
| CE | Cholesterol esters |
| CER | Ceramides |
| CL | Cardiolipidins |
| DG | Diacylglycerols |
| FA | Free fatty acids |
| LPC | Lysophosphatidylcholines |
| LPE | Lysophosphatidylethanolamines |
| MGDG | Monogalactosyldiacylglycerols |
| NAE | *N*-acylethanolamines |
| PA | Phosphatidic acids |
| PC | Phosphatidylcholines |
| PE | Phosphatidylethanolamines |
| PG | Phosphatidylglycerols |
| PI | Phosphatidylinositols |
| PS | Phosphatidylserines |
| SM | Sphingomyelin |
| TG | Triacylglycerols |
